# A Technical Comparison of Digital Frequency-Lowering Algorithms Available in Two Current Hearing Aids

**DOI:** 10.1371/journal.pone.0022358

**Published:** 2011-07-15

**Authors:** Hugh J. McDermott

**Affiliations:** The Bionic Ear Institute and The Department of Otolaryngology, The University of Melbourne, Victoria, Australia; Cuban Neuroscience Center, Cuba

## Abstract

**Background:**

Recently two major manufacturers of hearing aids introduced two distinct frequency-lowering techniques that were designed to compensate in part for the perceptual effects of high-frequency hearing impairments. The Widex “Audibility Extender” is a linear frequency transposition scheme, whereas the Phonak “SoundRecover” scheme employs nonlinear frequency compression. Although these schemes process sound signals in very different ways, studies investigating their use by both adults and children with hearing impairment have reported significant perceptual benefits. However, the modifications that these innovative schemes apply to sound signals have not previously been described or compared in detail.

**Methods:**

The main aim of the present study was to analyze these schemes'technical performance by measuring outputs from each type of hearing aid with the frequency-lowering functions enabled and disabled. The input signals included sinusoids, flute sounds, and speech material. Spectral analyses were carried out on the output signals produced by the hearing aids in each condition.

**Conclusions:**

The results of the analyses confirmed that each scheme was effective at lowering certain high-frequency acoustic signals, although both techniques also distorted some signals. Most importantly, the application of either frequency-lowering scheme would be expected to improve the audibility of many sounds having salient high-frequency components. Nevertheless, considerably different perceptual effects would be expected from these schemes, even when each hearing aid is fitted in accordance with the same audiometric configuration of hearing impairment. In general, these findings reinforce the need for appropriate selection and fitting of sound-processing schemes in modern hearing aids to suit the characteristics and preferences of individual listeners.

## Introduction

Two major hearing-aid (HA) manufacturers have recently introduced frequency-lowering sound processing schemes. Although these schemes are technically dissimilar, they are both intended for HA users who have relatively poor hearing at high frequencies. Lowering selected high-frequency components of sound has been shown to help some people with hearing impairment to perceive them [1], [2]. The perceptual benefits potentially include improved ability to resolve and discriminate between sounds as well as to detect them. As is well known, many people with sensorineural impairment have poorer hearing at high frequencies than at lower frequencies, as indicated by hearing sensitivity recorded on a pure-tone audiogram. In such cases, other aspects of auditory perception in addition to sound sensitivity are often affected. For example, frequency resolution, which is related to a listener' ability to separate a signal of interest such as speech from a background noise, is generally found to be poorer at frequencies having worse thresholds [3]. As a consequence, amplification by a HA may fail to enable every hearing-impaired listener to identify all sounds reliably, even though the audibility of those sounds is usually improved. Although various frequency-lowering schemes have been developed over several decades in attempts to address these problems, only two schemes are presently in widespread use.

The purpose of the present study was to measure and report the technical characteristics of these recently introduced digital frequency-lowering schemes. The first scheme was devised by Widex, a company based in Denmark, and is known as the Audibility Extender. It is a linear frequency transposition (LFT) scheme that has been reported to improve the understanding of some phonemes in speech, at least after training. For example, identification of fricative consonants increased by about 14 percentage points, on average, for eight adults after two months of use [2]. The second scheme, called SoundRecover, is available from Phonak, a company based in Switzerland. It is a nonlinear frequency compression (NLFC) scheme that was developed after promising perceptual results were reported for an experimental prototype [4]. Similar results have been published more recently [1]. They showed, for instance, that activation of the NLFC scheme increased mean scores by about 15 percentage points for 13 adults and 11 children in a test of plural-noun identification based on detection of a final /s/. The findings of the present study provide technical explanations for the perceptual benefits reported with use of both the LFT and NLFC frequency-lowering schemes.

### Frequency-lowering Techniques

The Widex LFT scheme functions by shifting components of sounds present within a source octave into a predetermined target octave [2]. As described in the Materials and Methods section, the settings chosen for the measurements reported below defined the source octave to encompass 2.5–5.0 kHz, and the target octave to be one octave lower (i.e., 1.25–2.5 kHz). In the LFT scheme, the contents of the source octave are analyzed periodically to identify a dominant spectral peak. The frequency of that peak is determined, and the amount of lowering is calculated such that the selected frequency is shifted down by one octave. Other frequency components in the source octave are shifted by an equal number of hertz. For example, if the peak frequency is 4 kHz, the extent of the downward shift is 2 kHz, resulting in the peak component being lowered to 2 kHz. At the same time, a source component at 5 kHz would be lowered by 2 kHz to 3 kHz. Note that, in general, only the frequency of the peak is shifted by exactly one octave. Consequently, it is possible that some components in the source octave would fall outside the target octave after shifting. For instance, in the above example a source component at 3 kHz would be lowered to 1 kHz. However, the signals resulting from the shifting process are filtered to ensure that they remain within the boundaries of the target octave. Thus a source component at 3 kHz would be discarded if the amount of lowering was 2 Hz (or any amount greater than about 1.75 kHz). After transposition, the contents of the target octave are mixed with any sound components already present in the same frequency region. Subsequently the usual processes of amplification, such as amplitude compression, are applied to the composite signal. An important characteristic of the LFT scheme is that the amount of frequency shifting generally varies over time in accordance with the frequency of the dominant peak in the source octave.

The Phonak NLFC scheme is based on different principles [4]. The processing has two adjustable parameters: the cut-off frequency and the frequency-compression ratio. For the present study, a cut-off of 2.3 kHz was chosen. This means that frequencies below 2.3 kHz are unaffected by the NLFC processing, whereas those above are compressed in frequency. The amount of lowering is progressive, such that frequencies much higher than the cut-off are shifted by a larger amount than frequencies only slightly above the cut-off. For example, the selected frequency-compression ratio of 1.7∶1 would result in a component at 1.7 oct above 2.3 kHz (i.e., 7.47 kHz) being lowered to a frequency 1 oct above 2.3 kHz (i.e., 4.6 kHz). The transfer function relating input to output frequencies is completely determined during fitting by selection of the above two parameters; it does not change in response to any signal characteristics. Signal components processed by the NLFC scheme do not overlap any other components present at the same time. Together with components below the cut-off frequency, signals that have been compressed in frequency are amplified and additionally processed as usual.

## Results

To obtain the measurements reported below, each HA was programmed according to the manufacturer' guidelines to provide an appropriate fitting for a sloping, severe-to-profound hearing loss (see Table 1). The input signals delivered to each HA comprised a sinusoid with slowly increasing frequency, a sequence of notes played on a flute, and four words chosen to contain many phonemes with dominant high-frequency components. Recordings from the Widex HA with and without LFT are available as Audio S1 and Audio S2 respectively, and the corresponding recordings for the Phonak HA are in files Audio S3 and Audio S4.

**Table 1:** Hearing threshold levels used to program the two hearing aids.

Frequency (kHz)	0.25	0.5	1	1.5	2	4	8
Hearing Threshold Level (dB HL)	50	60	70	80	90	100	100

### Measurements with Sinusoid

**Figure 1:**
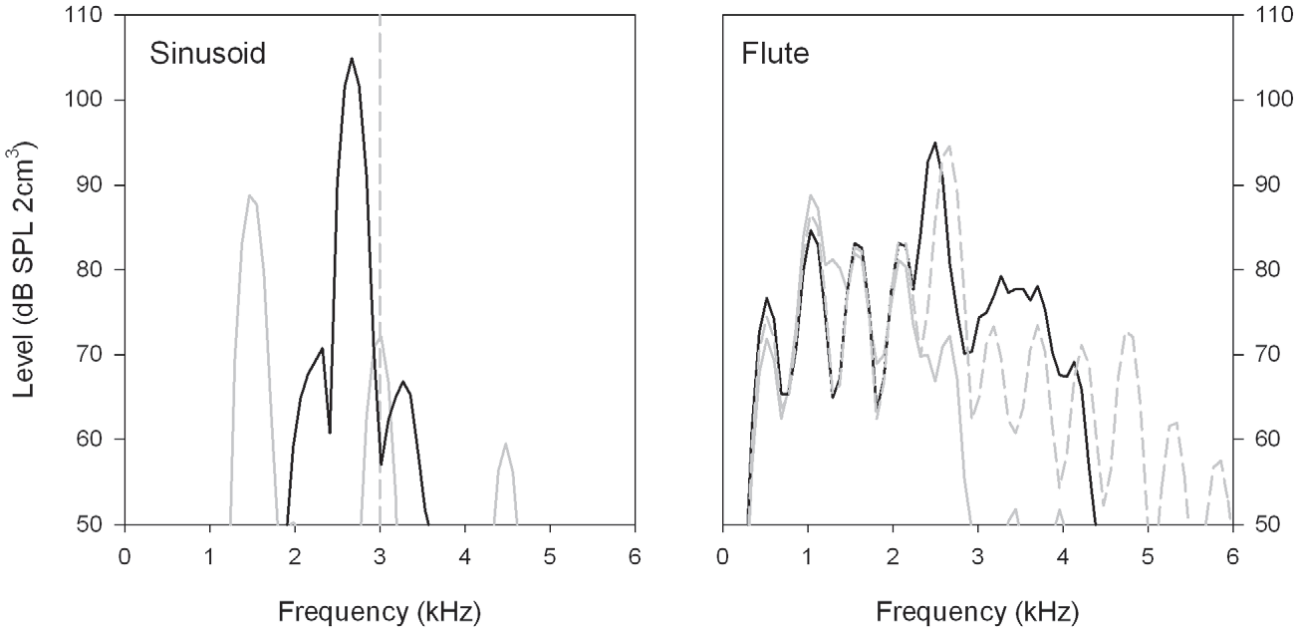
Output spectra of each hearing aid (HA) for inputs consisting of a sinusoid (left) and a tone produced by a flute (right). The sinusoid had a frequency of 3 kHz (vertical dashed line), corresponding to a brief portion of a sweep encompassing the frequency range 0.1–10 kHz. The flute note was C5 (fundamental frequency: 523.3 Hz). In both panels, the black line shows the spectrum from the Phonak HA with NLFC, and the gray line shows the spectrum from the Widex HA with LFT. The gray dashed line in the right panel shows the averaged spectrum from both HAs without frequency lowering.

Measurements on each HA with the frequency-lowering functions disabled confirmed, as expected, that the gains and output levels were very similar. Therefore, the spectrum for this condition shown in Figure 1 (dashed curve, right panel) is an average of the spectra obtained for each HA separately. The output of each HA for the swept sinusoid (not shown in the figures) conformed generally to expectations of the LFT and NLFC processing functions. For the Widex HA with LFT, the maximum output frequency was approximately 2.5 kHz, corresponding to a 1-oct lowering of the highest frequency in the source octave. For the Phonak HA with NLFC, the maximum output frequency was approximately 4.4 kHz, corresponding to an input frequency of about 6.8 kHz.

The short-term spectra for a brief portion of the swept sinusoid at which the input frequency to the HAs was around 3 kHz are shown in the left panel of Figure 1. The output of the Widex HA with LFT (gray) showed a high-level component at 1.5 kHz, which is 1 oct below the input frequency, as anticipated. Also evident were two lower-level components at 3 and 4.5 kHz which may have been at least partly artifacts of the processing. In comparison, the output of the Phonak HA with NLFC (black) had a single dominant peak at approximately 2.7 kHz, which is the output frequency expected for an input tone at 3 kHz with the selected parameter settings.

### Measurements with Flute Sounds

The right panel of Figure 1 shows the averaged spectra from each HA with and without frequency-lowering for one of the notes produced by the flute (C5). A 300-ms steady portion of this note was analyzed. As the fundamental frequency was approximately 523.3 Hz, and the signal waveform was essentially periodic, harmonics were present at frequencies of 1046.5, 1569.8, 2093.0 Hz, and so on. In both HAs, the first four harmonics produced almost identical outputs for the conditions with frequency-lowering disabled, and, for the Phonak HA, with NLFC enabled. With LFT, the same four frequency components were evident at similar levels, but the fifth harmonic (approximately 2.6 kHz) would have fallen into the source octave. As it was apparently identified as the dominant peak, it was shifted down by 1 oct to about 1.3 kHz. It therefore appeared between the second and third harmonics. There is evidence that a shift of the same amount (i.e., 1.3 kHz) was applied to the seventh harmonic (3.7 kHz) to produce an output component near 2.4 kHz. The unshifted fifth harmonic was also present in the output signal, but higher frequency components were at much lower levels. With NLFC, the fifth harmonic was shifted down to approximately 2.5 kHz, while the higher harmonics were shifted further and output at lower levels, corresponding to the relatively low level of harmonics above the fifth in the input signal.

### Measurements with Speech

**Figure 2:**
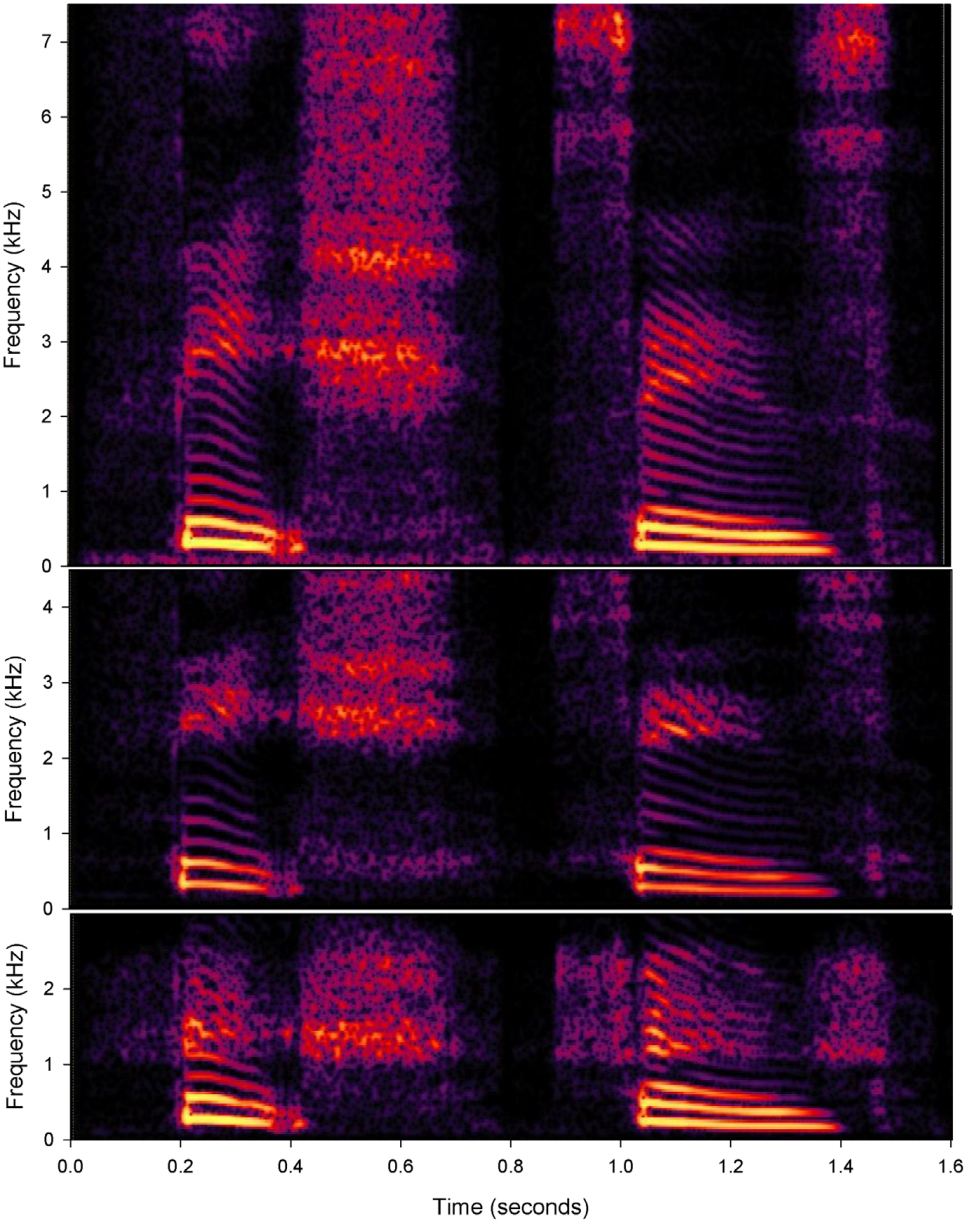
Spectrograms of the word sequence *fish, says* (top panel) and the corresponding outputs from each frequency-lowering hearing aid (middle: Phonak Nonlinear Frequency Compression; bottom: Widex Linear Frequency Transposition).

**Figure 3:**
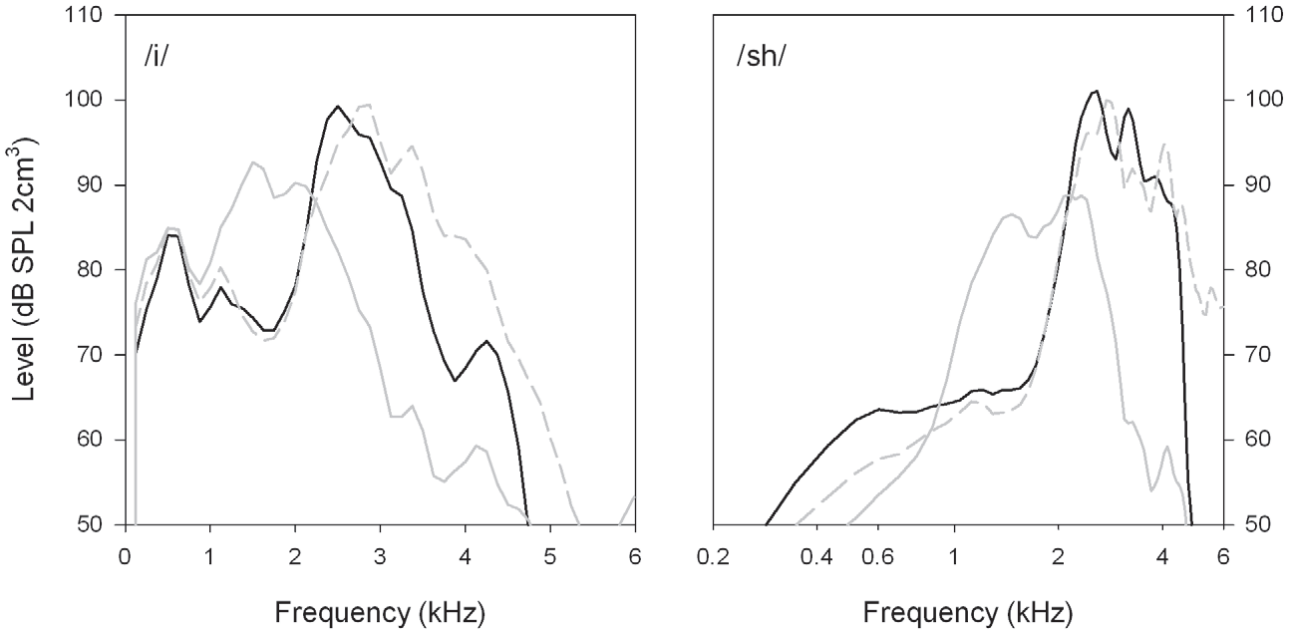
Output spectra of each frequency-lowering hearing aid for inputs consisting of the vowel /i/ (left) and the consonant /∫/ (right). Note that the abscissa in the right panel shows frequency on a log axis. Other details are as for Figure 1.

Figure 2 shows spectrograms of two of the words used in the tests (i.e., *fish*, *says*). The upper panel shows a spectrogram of the original signal, whereas the two lower panels show the outputs of the HAs with NLFC and LFT activated, respectively. The main effect of each type of processing is most evident in a comparison of a vowel sound, such as /i/ in approximately the 0.2–0.4 s portion of the spectrograms, and a consonant sound, such as /∫/ in the following portion up to about 0.7 s. Averaged spectra estimated from these two signals are shown in Figure 3. The spectra for /i/ (left panel) were obtained from a 50-ms steady portion near the vowel onset, whereas those for /∫/ (right) were obtained from a 200-ms steady portion within the consonant sound. As in Figure 1, the dashed curves show averages of the spectra for each HA with the frequency-lowering functions disabled.

For the vowel, a comparison of the spectra with the frequency-lowering functions enabled and disabled shows minimal effect for signal components near the first formant frequency (i.e., around 0.5 kHz). With LFT, components near the second formant (about 2.9 kHz) were lowered to approximately 1.5 kHz. The general effect of linear frequency transposition is clearly evident in that the shape of the spectrum in the source octave above 2.5 kHz with LFT disabled is similar to that with LFT enabled in the target octave below 2.5 kHz. With NLFC, the second-formant spectral peak was lowered to approximately 2.6 kHz, while higher-frequency components were lowered by progressively larger amounts.

For the consonant, the spectrum without frequency lowering shows two local peaks at about 2.8 and 4.1 kHz. With LFT, a peak is evident near 1.4 kHz, presumably corresponding to the lower input peak shifted down by 1 oct. There is also a second, relatively broad peak around 2.2 kHz which seems to have resulted from some combination of shifted and unshifted input components. With NLFC, the two input peaks were shifted to approximately 2.6 and 3.2 kHz, respectively. Some interpretations and implications of these results are discussed next.

## Discussion

In general, the above measurements are consistent with most expectations of the function of both LFT and NLFC processing. The effect of each scheme to reduce the bandwidth of output signals from the HAs is evident particularly in the spectrograms of Figure 2 and the spectra of Figure 3 (right). For LFT, the maximum output frequency was limited by the upper boundary of the target octave (i.e., 2.5 kHz), whereas that for NLFC was approximately 4.4 kHz. Note, however, that the output bandwidth of each HA is effectively adjustable by changing the parameter values of the frequency-lowering functions.

The tests with the swept sinusoid indicated that the Widex HA with LFT enabled produced at least two additional frequency components higher than the one expected from transposition of the input signal. Although this suggests some distortion in the LFT processing, it is likely that the levels of the extra components would be lower than the audibility threshold of a HA user with the audiogram used to program both devices (see Table 1). The tests with the flute sounds suggested that both HAs could provide accurate pitch information to listeners within the lowest four harmonics (including the fundamental) of the signal; see Figure 1 (right panel). Psychophysical studies have found that this frequency range tends to dominate listeners'perception of pitch for complex sounds [5]. Neither frequency-lowering scheme preserved accurate frequency differences between all of the harmonics. However, it seems plausible that the relatively small shift in the frequency of the fifth harmonic caused by NLFC processing would be less salient perceptually than the production by LFT of the component near 1.3 kHz. That component is not harmonically related to other components present in the input signal, and, given its comparatively high level, might reduce the ability of some hearing-impaired listeners to resolve the adjacent second and third harmonics.

The spectra obtained using the vowel sound also showed that some frequency ratios (or differences) between spectral peaks were altered by both LFT and NLFC; see Figure 3 (left). As expected, neither scheme changed frequencies near the first formant, but LFT shifted the peak near the second formant to about 1.5 kHz. In contrast, NLFC lowered that peak only slightly, with the result that it remained well within the overall range of second-formant frequencies for this vowel reported from measurements involving many different speakers [6]. Similar observations apply to the spectra of the consonant sounds (right panel). The relatively small effect of NLFC compared to LFT suggests that it might be easier for inexperienced listeners to adapt to the frequency-shifted signals, particularly when listening to speech, at least for the settings applied in the present tests.

In conclusion, both frequency-lowering schemes may provide perceptual benefits to HA users with hearing impairment at high frequencies. Although only one audiogram configuration was applied in the experiments, it is likely that the findings would be generally similar for other audiogram shapes, provided that they represented types of hearing impairment that would be suitable for fitting of either type of frequency-lowering HA. The technical test results suggest that the Phonak NLFC processing may preserve more details of the overall spectral shape than the Widex LFT scheme, at least for the selected signals and settings. However, the LFT scheme may be more suitable than NLFC for HA users with minimal usable hearing at frequencies above approximately 1.5–2 kHz. This is because the NLFC cut-off frequency is limited to a minimum setting of 1.5 kHz; thus, NLFC is unable to modify lower frequencies. In any case, selection of the optimum fitting for each HA user should depend ultimately on perceptual assessments, including tests of speech understanding in particular.

## Materials and Methods

The hearing aids used for the present study were the Widex mind440 m4-19 and the Phonak Naída V SP. Each was programmed to suit the audiogram shown in Table 1, based on default settings of the fitting software. This audiogram is well within each manufacturer' fitting guidelines for these HAs. Furthermore, it is close to the average audiogram of the subjects who participated in an evaluation of a prototype of the NLFC processing [4], and is within the range of audiograms of the subjects who participated in a published evaluation of the LFT scheme [2]. To ensure that the technical performance of each HA was not inadvertently affected by irrelevant aspects of the fitting, both HAs were programmed to match as closely as possible the gain and amplitude-compression characteristics recommended for this audiogram by the NAL-NL1 prescription [7]. In addition, signal-processing features such as feedback cancelation, noise reduction, and occlusion compensation were disabled, and an omni-directional microphone configuration was selected. These settings were not altered during measurements in which the LFT or NLFC schemes were either enabled or disabled. The selected settings of the frequency-lowering parameters for each HA are shown in Table 2.

**Table 2:** Settings of the frequency-lowering schemes in the two hearing aids.

Widex LFT		Phonak NLFC	
Source octave	Target octave	Cut-off frequency	Compression ratio
2.5–5.0 kHz	1.25–2.5 kHz	2.3 kHz	1.7∶1

Output signals were recorded from each HA in each condition for three types of input signal: (1) a sinusoid swept from 0.1 to 10 kHz logarithmically over 10 s; (2) a succession of notes played on a flute; and (3) speech, comprising four monosyllabic words recorded by a female speaker. The average level of all signals was 65 dB SPL. The sounds were delivered to each HA in a Brüel & Kjær Type 4222 anechoic test chamber, and the output signals were recorded via a 2-cm^3^ coupler for later analysis using Adobe Audition 3.0 software. The swept sinusoid, which was used to verify the function of each HA with and without each frequency-lowering scheme, was passed through a low-pass filter with frequency response similar to the long-term average speech spectrum [8] before delivery to the HAs. This ensured that the level across frequency was well within the range at which optimal processing could be expected for each HA. The flute sounds were included to investigate the potential effects of frequency lowering on musical pitch, and comprised a sequence of notes ranging from G4 to G5 (i.e., fundamental frequencies 392–784 Hz). The words in the speech material (*thatch, fish, says, verge*) were chosen to include eight different fricative or affricate consonants that are common in English and contain important acoustic components at relatively high frequencies.

The audio signals recorded from the HAs were sampled at 44.1 kHz with 16-bit resolution. The spectra shown in Figures00201 and 3 were obtained using a 512-point Fast Fourier Transform (FFT) preceded by a Blackman-Harris windowing function. The spectrograms shown in Figure 2 were obtained using a 256-point FFT after the original signals had been down-sampled to 16 kHz.

## Supporting Information

Audio S1 **Sound recording from the Widex hearing aid (HA) with the Linear Frequency Transposition (LFT) function disabled.** The input signals were four monosyllabic words (*thatch, fish, says, verge*), a sequence of notes played on a flute, and a swept sinusoid (0.1–10 kHz).
(WAV)

Audio S2 **As for** **Audio S1** **, but with LFT enabled.**
(WAV)

Audio S3 **As for** **Audio S1** **, but for the Phonak HA with the Nonlinear Frequency Compression (NLFC) function disabled.**
(WAV)

Audio S4 **As for** **Audio S1** **, but with NLFC enabled.**
(WAV)
